# Long-Acting Antipsychotic Injections: Prescribing Practices Across BCUHB

**DOI:** 10.1192/bjo.2025.10533

**Published:** 2025-06-20

**Authors:** Laura Robbins, Vinila Zachariah, Opeyemi Ikuewumi, Raj Sambhi

**Affiliations:** 1 BCUHB, Wrexham, United Kingdom.; 2 BCUHB, Rhyl, United Kingdom.; 2 BCUHB, Llanfairfechan, United Kingdom

## Abstract

**Aims:** Our aims for this project were to examine how long-acting antipsychotics are prescribed in the various teams (both inpatient, community and specialist services) across BCUHB, with a view to identify any emerging trends, and to compare this with data on efficacy and cost-effectiveness obtained via a systematic search of the available literature.

**Methods:** Data on depots prescribed across BCUHB was provided to us by the mental health pharmacy team for the year April 2023–March 2024. We extracted our points of interest from this data and demonstrated this graphically using Microsoft Excel.

We also completed a literature search of Ovid, Cochrane Library and Google Scholar on the topic, to identify relevant systematic reviews which included studies comparing depot antipsychotics head-to-head. This returned 1500 articles, of which 15 were shortlisted by title relevance, and 4 included following full-text analysis.

**Results:** According to the available research, there is no demonstrated clear superiority in efficacy of specific long-acting antipsychotics. The data on cost-effectiveness was somewhat conflicting; in that risperidone was found in a recent systematic review to be the most cost-effective in most studies apart from included UK studies; but that also paliperidone was more cost-effective than the typical antipsychotics. Our data showed that the three most commonly prescribed in BCUHB are typical antipsychotics, and interestingly, the unit price per depot for paliperidone in BCUHB was significantly higher than any other.

**Conclusion:** ‘Cost-effectiveness’ in the systematic review we looked at was defined by QALYS (‘one year of life in perfect health’). To look at the BCUHB ‘price per depot’, you may, incorrectly, assume that prescribing paliperidone would be a waste of money (with it being 173 times more expensive than the highest dose of the cheapest depot available). This suggests that use of paliperidone may make cost-savings in the longer-term, for example, in preventing admissions to hospital which are costly. In BCUHB, paliperidone is commonly prescribed to patients with learning disabilities, but is not a commonly prescribed depot amongst general adult groups (either inpatient or community).

There is limited guidance on choice of antipsychotic depot and given the absence of significant differences in their efficacy, it is generally down to clinician choice, taking into account patient preferences and drug tolerability profiles. As mentioned, cost does not equal cost-effectiveness and having an awareness of this may influence local guidance and decision-making.

